# Eye acupuncture for pain conditions: a scoping review of clinical studies

**DOI:** 10.1186/s12906-021-03272-8

**Published:** 2021-03-23

**Authors:** Yuan Chi, Jürgen Barth, Mei Wang, Nicola Robinson, Zan-Hua Li, Jian-Ping Liu

**Affiliations:** 1 Centre for Evidence-Based Chinese Medicine, Beijing University of Chinese Medicine, 11 Bei San Huan Dong Lu, Chaoyang District, Beijing, 100029 China; 2 Institute for Complementary and Integrative Medicine, University Hospital Zurich and University of Zurich, Zurich, Switzerland; 3 School of Basic Medicine, Liaoning University of Traditional Chinese Medicine, Shenyang, China; 4 Institute of Health and Social Care, London South Bank University, London, UK; 5 Department of Pain Management, The Second Hospital of Shandong University, Jinan, China; 6 Institute of Integrated Traditional Chinese Medicine and Western Medicine, Guangzhou Medical University, Guangzhou, China

**Keywords:** Pain relief, Traditional Chinese medicine, Eye acupuncture, Clinical studies, Scoping review

## Abstract

**Background:**

Chinese eye acupuncture, focuses on treating different diseases by applying needle stimulation around the orbit of the eye. Since 1970, it has been used in China for the management of pain-related disorders. This scoping review systematically collected clinical studies on the use of eye acupuncture to treat pain conditions and identify any adverse events.

**Methods:**

Six databases including PubMed, the Cochrane Library, CNKI, VIP, Wan Fang Data and SinoMed were searched from 1970 to March 2019. Randomized controlled trials (RCTs), clinical controlled trials (CCTs) and case series on eye-acupuncture for pain conditions meeting the inclusion criteria were identified. Data were extracted on patients, interventions, details of eye acupuncture, control treatments and outcomes.

**Results:**

Searches identified 81 clinical studies and a trend demonstrating an increasing number of published studies. All studies were conducted in China and published in Chinese. These included, 45 (55.6%) RCTs, 5 (6.2%) CCTs, and 31 (38.3%) case series, treating 7113 patients with 44 different pain-related diseases or symptoms. The most frequently reported conditions were headache (18, 16.2%), acute lumbar pain (7, 6.3%) and lumbar disc herniation (7, 6.3%). Treatment using small needles (φ0.25 × 13 mm), retained ≤30 min, needling the horizontal outer orbital edge and the avoidance of manipulation during treatment were the most frequent descriptions of the interventions used. Eye acupuncture was used alone in about half of the studies and of the remaining studies it was combined with other treatment. All studies suggested some beneficial effects including: pain relief, improved quality of life and mental health, and 18 (22.2%) adverse events.

**Conclusion:**

Eye acupuncture, predominantly studied in China, may be a promising intervention for managing diverse pain conditions. However, given the variety of study designs and reported treatment outcomes, conclusions about the evidence for eye acupuncture for specific conditions are not possible at this stage.

**Supplementary Information:**

The online version contains supplementary material available at 10.1186/s12906-021-03272-8.

## Background

For pain management, both the 2016 Centers for Disease Control and Prevention (CDC) guideline [1] and the 2017 Canadian Guideline for Opioid for Chronic Non-Cancer Pain [2] recommended non-opioid pharmacotherapy and non-pharmacologic therapy. The role of opioids in the treatment of chronic pain has increasingly come into question due to the risk of addiction, brain damage and overdose-induced death [3–6] and has insufficient evidence on its benefits [2, 7]. Non-pharmacotherapy options to treat chronic pain include: patient education, cognitive-behavior therapy and complementary and alternative therapies [8]. Body acupuncture, as part of Traditional Chinese Medicine (TCM), has become increasingly used for pain relief worldwide [9, 10], given its beneficial effects for treating pain and disability [11] and provides a potential opportunity for integration into patient-centered care [12].

Eye acupuncture is an understudied intervention and involves fine-needle acupuncture, and may also include embedding catgut at acupoints and acupressure applied around the orbit of the eye [13]. Based on the TCM theory of the relationship of the eyes to the brain, viscera, and meridians [14, 15], this therapy originated in Liaoning province, China and was developed by an acupuncturist Jing-Shan Peng in 1970 [16]. Clinical practice, clinical experimental and observational studies [17, 18] and animal experiments [19–22] have demonstrated possible mechanisms in regulating the nervous system, blood measures and molecular-level expression.

Eye acupuncture is much less complicated compared to traditional body-acupuncture as it only employs 13 acupoints, namely lung, large intestine, kidney, bladder, upper jiao, liver, gallbladder, middle jiao, heart, small intestine, spleen, stomach, and lower jiao [16]. Except for the three acupoints that represent the regions of the body (upper jiao, regions above the horizontal line on the diaphragm, included upper limbs; lower jiao, regions below the horizontal line on umbilicus; and middle jiao, the remainder), the remaining ten acupoints are all named according to the related viscera. These 13 acupoints are located at 2 mm from the outer side of the orbit in the eight areas divided equally by four pupil-centered lines. An additional figure presents the location of the 13 acupoints (see Additional File 1). A previous anatomical study has demonstrated that eye acupuncture needles are inserted through the skin, superficial fascia, deep fascia to reach orbicularis oculi muscle (avoid touching the periosteum). The superficial fascia underneath each acupoint area is in somatosensory nerves and the vascular network is enclosed by visceral sensory nerve endings [23].

A scoping review is a relatively new evidence synthesis method for identifying knowledge gaps, examining the body of associated literature, clarifying concepts or investigating the conduct of research [24]. Data can be used to inform future clinical trials in order to prioritize conditions or treatment regimens. Scoping reviews can also be precursors to conducting systematic review and therefore may be an appropriate approach to explore existing evidence for eye acupuncture and its use in reducing pain.

The aim of this scoping review was to identify clinical studies and reports on eye acupuncture with the aim of informing practitioners, patients and researchers about the use of this emerging intervention for pain relief.

## Methods

### Eligibility criteria

This scoping review used the Preferred Reporting Items for Systematic Reviews and Meta-Analyses (PRISMA) and the PRISMA Extension for Scoping Reviews (PRISMA-ScR) guidelines [25, 26].

### Types of studies

Relevant clinical studies included randomized controlled trials (RCTs), controlled clinical trials (CCTs) and case series, regardless of language, publication form or publication status were identified. Case reports which could supply any additional valuable information were also collected and are reported in the appendix but were not included in the overall analysis.

### Types of patients

Patients with any pain-related diseases/symptoms were eligible. Both primary and associated symptoms were included. Studies were omitted if they failed to mention patients’ pain symptoms even though the disease might involve pain.

### Types of interventions

Experimental interventions involving needle insertion, laser stimulation or transcutaneous electrical stimulation at the 13 acupoints, described as eye acupuncture, were eligible. Studies applying eye acupuncture therapy alone or in combination with other treatment were all considered eligible. For RCTs and CCTs, all types of control interventions were eligible.

### Types of outcome measures

Studies were included if they reported outcomes of any pain-related symptom as either a primary or a secondary symptom.

### Literature search

Two English and four Chinese electronic databases were searched from 1970 or from their inception through to 9 March 2019. An additional file provides the complete search strategy and results for the English database PubMed and the Chinese database CNKI (see Additional File 2).

Databases were:

- PubMed (http://www.ncbi.nlm.nih.gov/pubmed/) (1970 to 2019)
- The Cochrane Library (Issue 3, 2019) (http://www.cochranelibrary.com/)
- China Network Knowledge Infrastructure (CNKI) (http://www.cnki.net/) (1970 to 2019)
- Chinese Scientific Journals Database (VIP) (http://www.cqvip.com/) (1989 to 2019)
- Wan Fang data (http://www.wanfangdata.com.cn/index.html) (1985 to 2019)
- SinoMed (http://www.sinomed.ac.cn/) (1978 to 2019)

The reference lists of included studies were scrutinized to identify possible additional relevant studies.

One author (YC) first screened titles and abstracts using NoteExpress 3.2.0.7103. Potentially eligible articles were then read in full by YC to determine whether they met the eligibility criteria.

### Data extraction and analysis

One author (YC) extracted the data into a pre-designed electronic form using Microsoft Excel 2016 and crosschecked the data for duplications.

We designed our data extraction form followed the PRISMA and the PICO framework [27]. The extracted information included bibliometric data (published year, reference type, origin, funding, published language, journal, study type), patients (sample size, gender, age, disease/symptom), interventions (acupoints, comparisons, characteristics of needles and operations) and outcomes (symptom reduction, pain scales, adverse events, follow up).

These data were then analyzed descriptively by calculating frequencies, and percentages. Graphical representations of the data were created in Microsoft Excel 2016 and Microsoft Power BI. The flowchart was created using Microsoft Visio 2016. All the figures were imported in and processed via Adobe Photoshop (CC 2017).

## Results

### Literature search

**Fig. 1:**
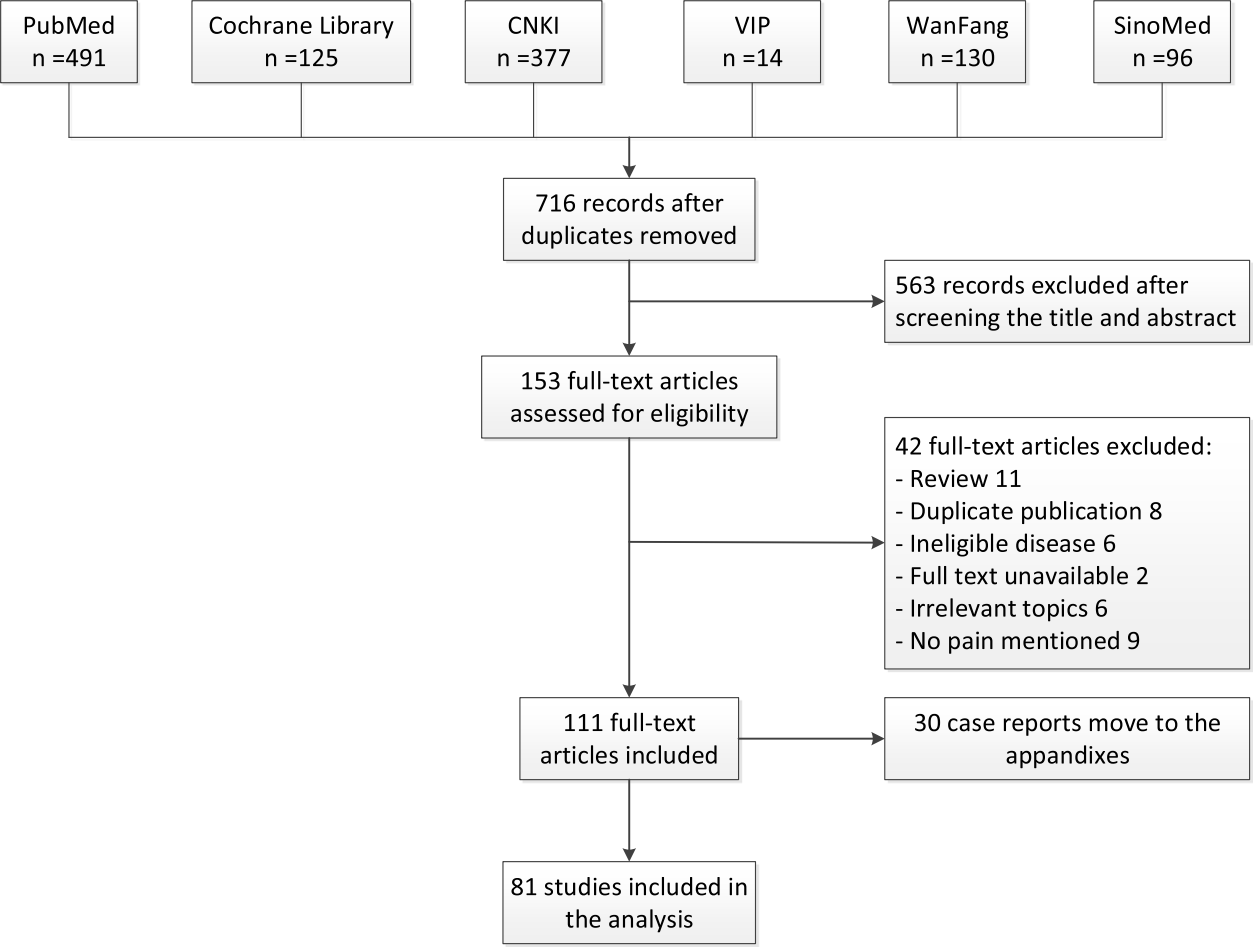
Flowchart of the literature search

A total of 940 records were retrieved from the literature search. Any duplicates were excluded, and the remaining studies were assessed for eligibility. The flowchart shows the selection process (Fig. 1). The review was conducted on 81 full-text articles [28–108]. Additionally, 30 related case reports were identified, but this information is presented in an additional file (see Additional File 3).

### Bibliometric information

The 81 studies included 45 (55.6%) RCTs, 5 (6.2%) CCTs, and 31 (38.3%) case series. There was an increasing overall trend in the number of publications year by year. An additional figure illustrates the year of publications in terms of study type (see Additional File 4), and the percentage which were RCTs. The earliest RCT [71] was published in 1995. Since 2008, RCTs have taken over as the dominant study type from case series.

The 81 analyzed studies, all conducted in China, originated from 20 different provinces. The 81 studies consisted of, 64 journal articles (79.0%), 14 master’s theses (17.3%) and three conference papers (3.7%). The 64 journal articles appeared in 33 different journals, with the journal of *Chinese Acupuncture & Moxibustion* (9, 14.1%) and the *Journal of Clinical Acupuncture and Moxibustion* (8, 12.5%) being the most prevalent.

### Patients

In total, 7113 patients participated in the 81 studies. For trials (RCTs *n* = 45 and CCTs *n* = 5), study sample sizes ranged from 24 to 248 (median: 60; average: 84.1). Among the 81 studies, a total of 74 (91.4%) studies reported gender (male: 2976, 47.6%; female: 3272, 52.4%) and 66 (81.5%) studies reported age, with a range between 9 to 85 years. The duration of disease was reported in 60 (74.1%) studies with a range of 1 h to 34 years. The treatment duration was reported in 59 (72.8%) studies and ranged from 1 day to 4 months, of which, 4 (6.8%) studies reported patients cured with a one-off treatment, and 56 (94.9%) studies applied treatment within a 2 months period.

**Fig. 2:**
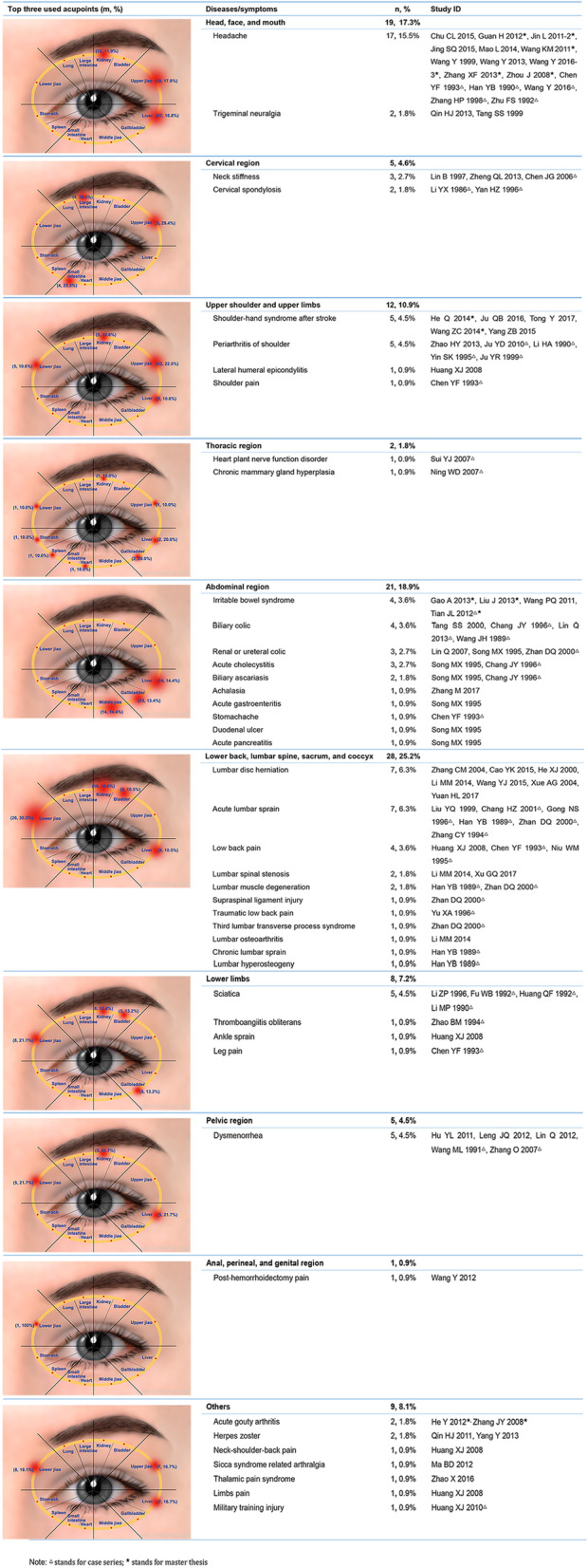
Conditions and used acupoints. m = frequency the acupoints used; % = m/total frequency of all the acupoints used in that condition

The 81 studies covered 44 different diseases/symptoms. There were 108 conditions in total as there were studies that contained more than one condition. The conditions were classified according to the International Association for the Study of Pain (IASP) Classification of Chronic Pain to the IASP’s Axis 1 (regions) coding scheme for chronic pain diagnoses [109] which clearly demonstrates the results obtained [Fig. 2]. Headache (18, 16.2%) was the most frequently reported, followed by acute lumbar sprain (7, 6.3%), lumbar disc herniation (7, 6.3%), shoulder-hand syndrome after stroke (5, 4.5%), periarthritis of shoulder (5, 4.5%), sciatica (5, 4.5%) and dysmenorrhea (5, 4.3%).

### Interventions

#### Selection of acupoints

All studies reported their selection of acupoints. Their use was shown pictorially. The three most frequently used acupoints in each region of the orbit are given in Fig. 2. Details of acupoints used for each condition are shown in an additional figure (see Additional File 5).

#### Treatments and comparators

In terms of treatments used in the 81 studies, 44 (54.3%) used eye acupuncture alone and the others used eye acupuncture combined with other treatments. In the 50 trials (RCTs and CCTs), 55 eye acupuncture related comparisons were reported. Details of the treatment for each comparison are shown in an additional table (see Additional File 6). The treatments were first summarized in seven categories. In a second step for each category, subgroups were displayed by the intervention in the control group. Twenty-two comparisons (40.0%) applied eye-acupuncture alone as an intervention in the treatment group; correspondingly, non-pharmaceutical traditional Chinese therapy (17, 30.9%) and conventional medicine (14, 25.5%) were the most commonly used control treatments.

#### Characteristics of the needles

**Fig. 3:**
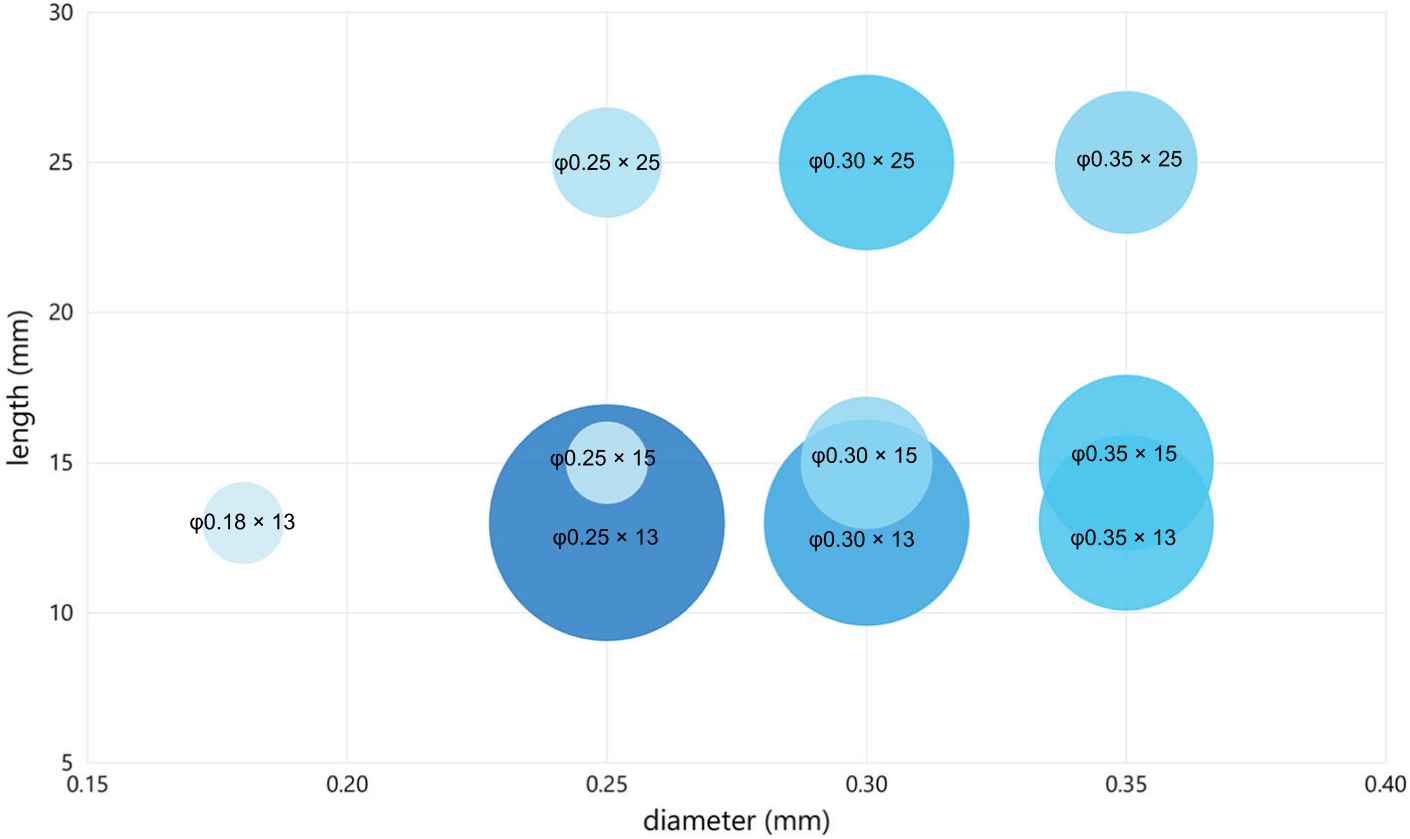
Size of acupuncture needle

Of the 81 studies, 68 (84.0%) referred to the characteristics of acupuncture needle used, including brand/material (39, 48.1%), length (64, 79.1%) and diameter (55, 67.9%). The details of needle size are shown in (Fig. 3). It is important to mention that since the measuring unit of acupuncture needle varies, such as *cun* or *fen* for length and *hao* for diameter, a conversion has been done to generalize the parameters in millimeter (mm). The most frequently used needles were stainless steel acupuncture needles in φ0.25 mm × 13 mm.

#### Needling techniques

**Fig. 4:**
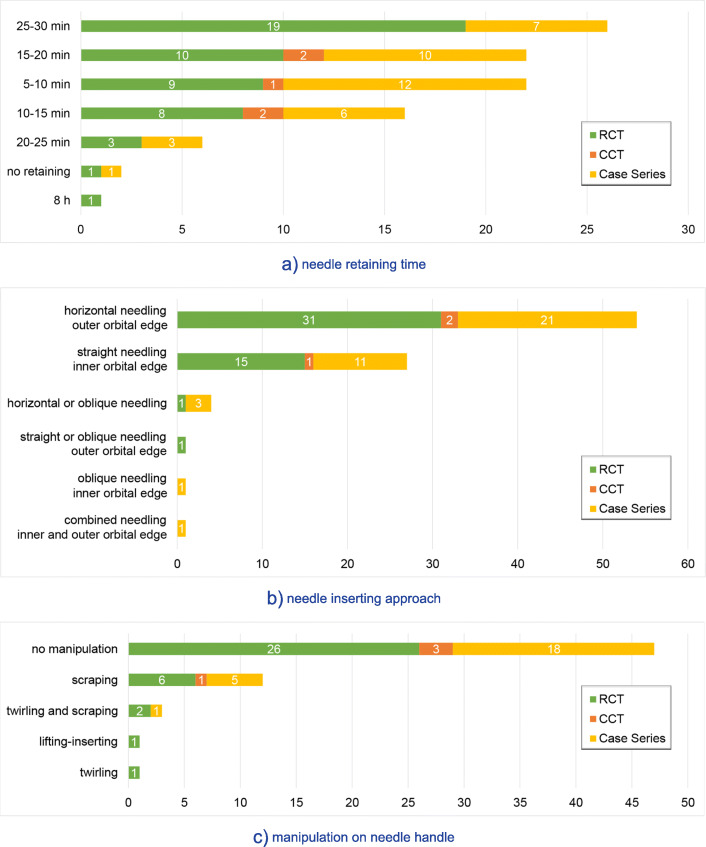
Needling techniques. Abbreviations: RCTs: randomized controlled trials, CCTs: controlled clinical trials

The basic techniques of eye-acupuncture needling varied between studies. A total of 78 studies mentioned the needle retention time, ranging from “not retaining the needle” to 8 h, with 30 min (21 studies, 26.9%) being the most frequently used duration. Of the 69 studies (85.2%) that described the approach to needle insertion, 54 studies (78.3%) used ‘horizontal needling outer orbital edge’, 27 studies (39.1%) used ‘straight needling inner orbital edge’, and 17 studies (24.6%) used both simultaneously. Of the 66 studies (81.5%) that mentioned whether the needle was manipulated or not, 47 studies (71.2%) made it explicit not to exert any manipulation of the needle during the treatment. Two studies did not specify the kind of manipulation. For the remaining 45 studies, the details of each needling technique are shown in Fig. 4.

#### Assisted body exercise

In 32 conditions (29.6%), patients were asked to exercise (from slight movement ranges and increase the motion gradually) their affected body parts during eye-acupuncture treatment. This applied to more than half of the conditions and related to the three musculoskeletal regions: ‘lower back, lumbar spine, sacrum, and coccyx’ (17, 63.0%), ‘upper shoulder and upper limbs’ (6, 54.6%) and ‘cervical region’ (3, 60%).

### Treatment effects and side effects

Of the 18 studies (22.2%) [33, 35, 37, 41, 42, 49, 62, 65, 79, 81, 83, 86–88, 91, 99, 102, 107] reporting outcomes on adverse events, seven studies [37, 49, 65, 79, 83, 88, 91] found no adverse events, and four studies [35, 62, 81, 87] reported patients having slight bleeding when the needle was withdrawn. The remaining seven studies reported adverse events from eye acupuncture as well as events from other interventions during the treatment, which are shown in Table 1. The effects following an adverse event in those receiving acupuncture were reported to have disappeared after rest. Follow-ups were reported in 15 (18.5%) studies, with follow up periods ranging from 2 days to 12 months in eight of the case series and 3 months to 6 months in seven RCTs.

**Table 1:** Details of adverse events of eye acupuncture for pain conditions

Study (pain condition)	Groups	Adverse events
Zhou J 2008 [107] (migraine)	I: eye acupuncture C1: eye acupuncture + body acupuncture C2: body acupuncture	I: — C1: fainting (2, 6.7%) C2: fainting (1, 3.3%)
He Y 2012 [41] (acute goutyarthritis)	I: eye acupuncture C: body acupuncture	I: dizziness, chest distress, nausea (1, 3.4%) C: —
Zhang XF 2012 [102] (migraine)	I: eye acupuncture C1: eye acupuncture + body acupuncture C2: flunarizine hydrochloride	I: fainting (1, 3.3%) C1: fainting (1,3.3%) C2: dizziness (1, 3.3%)
Zhang JY 2008 [99] (acute gouty arthritis)	I: eye acupuncture C: Chinese herbal medicine + Indomethacin	I: abdominal discomfort (1, 3.6%) C: abdominal discomfort (3, 10.3%)
Hu YL 2011 [42] (primary dysmenorrhea)	I: eye acupuncture C: ibuprofen	I: — C: dizziness, headache, rash, nausea, vomit (9, 18%)
Chu CL 2015 [33] (menstrual headache)	I: eye acupuncture + Chinese herbal medicine C: Chinese herbal medicine	I: diarrhea (3, 9.4%); ecchymoma (1, 3.1%); nausea (1, 3.1%) C: fainting (1, 3.1%); diarrhea (1, 3.1%); ecchymoma (4, 12.5%)
Wang Y 2012 [86] (post-hemorrhoidectomy pain)	I: eye acupuncture C: bucinperazine injection	I: analis edema (2, 4.2%); urinary retentron (1, 2.1%); nausea (1, 2.1%) C: analis edema (1, 2.1%); urinary retentron (2, 4.2%); nausea (2, 4.2%)

*T* treatment group, *C *control group

In total, 77 studies reported an overall response rate (ORR) (87–100%, median = 96.7%). ORR stands for the sum of cure rate, significant effective rate, and improvement rate, however, since the definitions of ORR varied from diseases and studies, further analysis on ORR was not feasible. All studies reported a positive result for the eye acupuncture intervention, while two three-arm RCTs [45, 107] showed that a combination of eye acupuncture and body acupuncture had a higher ORR compared to eye acupuncture alone. The authors explained that eye acupuncture was more effective in treating diffuse pain, while body acupuncture was more effective for treating a local pain. For treating complex pain, when two acupuncture techniques were used, outcomes were further improved. For 57 conditions (52.8%), patients acquired an immediate analgesic effect after acupuncture, however, 38 (66.7%) of these conditions were from case series studies. In the 32 pain conditions which encouraged adjunctive exercise, 26 (81.3%) reported immediate analgesic effects. All the 81 studies presented data showing an improvement in pain relief, including the degree of pain relief, time spent without pain, relief of tenderness, reduction in persistent periods of pain, numbers of attacks, improvements in the range of motion of joints and TCM syndrome score. Apart from pain symptoms, treatment effects were also estimated by recurrences (9, 8.1%), changes in quality of life (including: activities of daily living (13, 11.7%), ability to work or study (20, 18.0%) and social life (2, 1.8%)), and mental health (2, 1.8%). No studies reported on economic outcomes. Twelve international instruments were used in 23 studies and the Visual Analogue Scale (VAS) was the most often used. Fifty-eight papers did not use a standardized scale.

### Funding sources

Seven studies [28, 49, 92, 94, 100, 105] reported funding sources and were all RCTs published in the years 2015 to 2018. All funding came from the government, including one national project, three provincial, and three municipal sources.

## Discussion

### Main findings

A total of 81 clinical studies using eye acupuncture were identified and an increasing publication trend from 1977 to 2019, culminating in 45 (55.6%) RCTs, 5 (6.2%) CCTs, and 31 (38.3%) case series, with the RCT being the main study type after 2008. The analyzed studies had a total of 7113 patients with 44 with different pain-related diseases/symptoms, representing both genders and covering all ages. The most commonly included conditions were headache, acute lumbar pain, lumbar disc herniation, shoulder-hand syndrome after stroke, periarthritis of shoulder, sciatica and dysmenorrhea. The most common needling techniques were, using small stainless-steel acupuncture needles (φ0.25 mm × 13 mm), retained for no more than 30 min, needling horizontal outer orbital edge and avoiding manipulating during the treatment. Additional body exercises were applied in some studies for treating musculoskeletal pain, most of which showed immediate analgesic effects.

Eye acupuncture was used alone in 44 (54.3%) studies and in 22 (40%) studies as a trial comparator, and the others used eye acupuncture combined with other treatment. Only 18 (22.22%) studies reported on the outcome of adverse events. Outcomes were measured by multi-dimensional scales of pain symptoms, recurrence, quality of life, and mental health, with the VAS being the most frequently used instrument.

### Limitations of this analysis

Our study has three main limitations. First, since the taxonomy of disease is developing and not fixed [110], it caused inevitable overlapping among the classification of pain. Second, all the included studies were published in China, and nearly one third (28.9%) of the RCTs were conducted as a master’s thesis, and funding was not mentioned. The quality of such studies may, therefore, be questionable, because procedures such as randomization, recruitment or measurement may not have been appropriately applied. Third, this report is unable to present information on the long-term outcomes and patients’ quality of life due to lack of data on follow-up in the included studies.

### Implications for research

The number of RCTs identified, indicate that this field already has a history and is continuing to develop. Case series will provide some evidence on effectiveness in a real-world setting but this cannot be used as evidence of effectiveness [111].

Despite the wide-ranging duration of diseases, nearly all studies reported a treatment intensity with a duration shorter than 3 months and 29 studies used one-off eye acupuncture treatment. Although the included studies have suggested that those patients with a shorter duration of the disease were more likely to receive less intense, short periods of treatment [68, 80, 95, 108] we found no evidence to confirm this assumption. The treatment intensity also depends on the type of acupuncture treatment and the patient’s ability to quickly recovery [112]. If eye acupuncture therapy was acceptable and able to contribute to shorter treatment time in general, it could reduce patients’ health care costs as well as social burden [113]. Unfortunately, no economic data were found in this analysis.

### Implications for practice

The choice of eye-acupuncture needle and needling techniques are more important to consider in terms of safety rather than regarding the effectiveness of the intervention There is a rich blood capillary network around the orbit and withdrawing the needle there may be the possibility of slight bleeding [114]. So small needles inserted horizontally outside the orbital without any manipulation could lower such risks, as well as the needling time of no more than 30 min, which is widely accepted around the world in body acupuncture.

It is known that exercise interventions could alter musculoskeletal pain memories [115], including anxiety and fear-avoidance [116]. Combining eye-acupuncture and appropriate exercise can enhance outcomes: on the one hand, eye-acupuncture can relieve pain directly; on the other hand, eye-acupuncture may enhance the efficiency of doing exercises. Since eye-acupuncture acupoints all located around the orbit, patients are able to exercise while retaining the needle.

## Conclusions

Eye acupuncture has been substantially studied in China and seems to be a promising intervention used in the management of diverse pain conditions, and appeared to have no specific reported adverse events compared to other kinds of treatment. However, given the variety of study designs and reported treatment outcomes, conclusions about the evidence for eye acupuncture for specific conditions are not possible at this stage.

## Supplementary Information

**Additional file 1.**

**Additional file 2.**

**Additional file 3.**

**Additional file 4.**

**Additional file 5.**

**Additional file 6.**

## Data Availability

The data used to support the findings of this study are included within the article and the supplementary information files. Additional data will be provided on a reasonable request to the first author.

## Abbreviations

CDC: Centers for Disease Control and Prevention
TCM: Traditional Chinese Medicine
PRISMA: Preferred Reporting Items for Systematic Reviews and Meta-Analyses
RCTs: Randomized controlled trials
CCTs: Controlled clinical trials
VAS: Visual analogue scale
