# Supplementary figures and images for: Eye acupuncture for pain conditions: a scoping review of clinical studies
Source: BMC Complement Med Ther. 2021 Mar 23;21:101. doi: 10.1186/s12906-021-03272-8 (PMC7989101; doi:10.1186/s12906-021-03272-8)

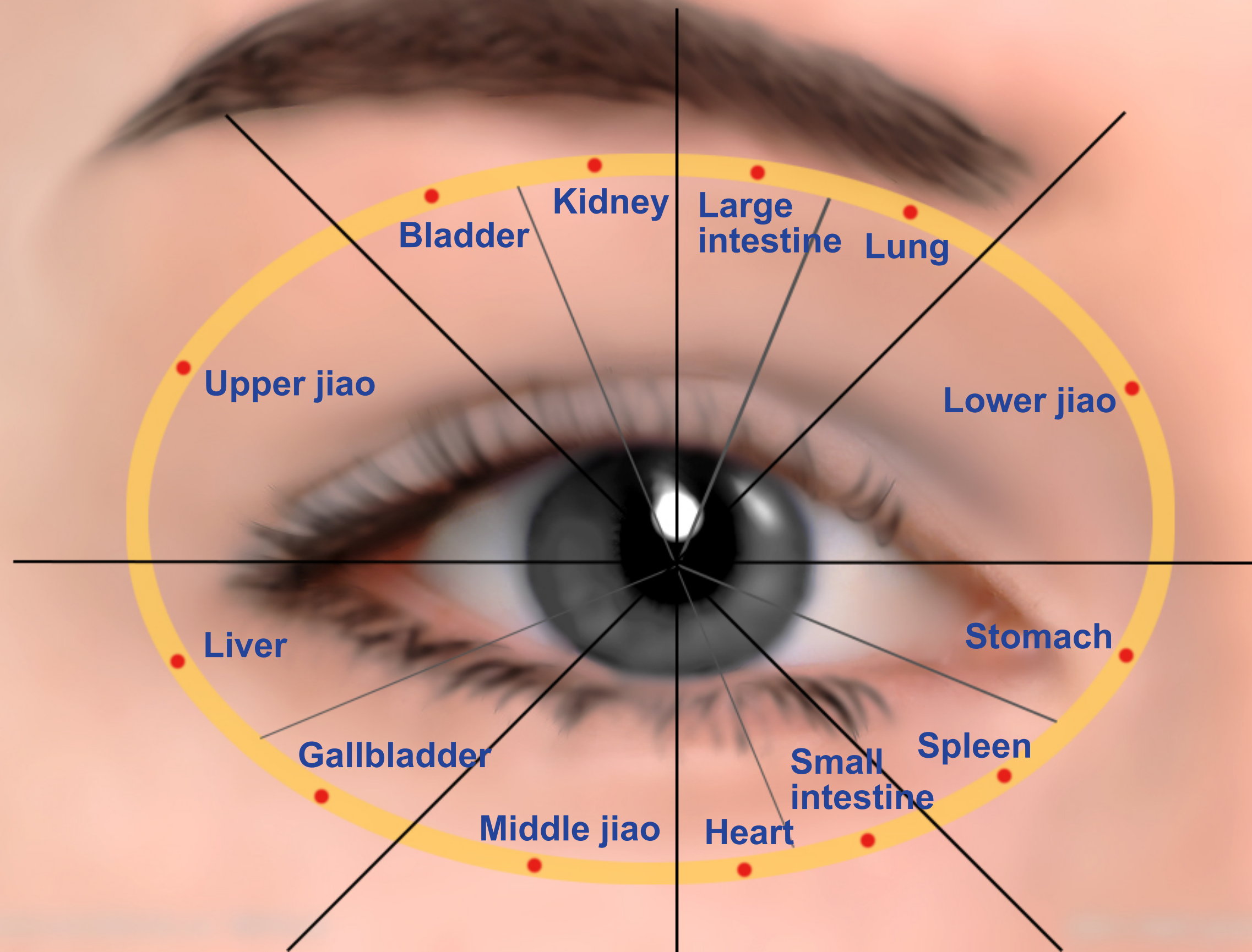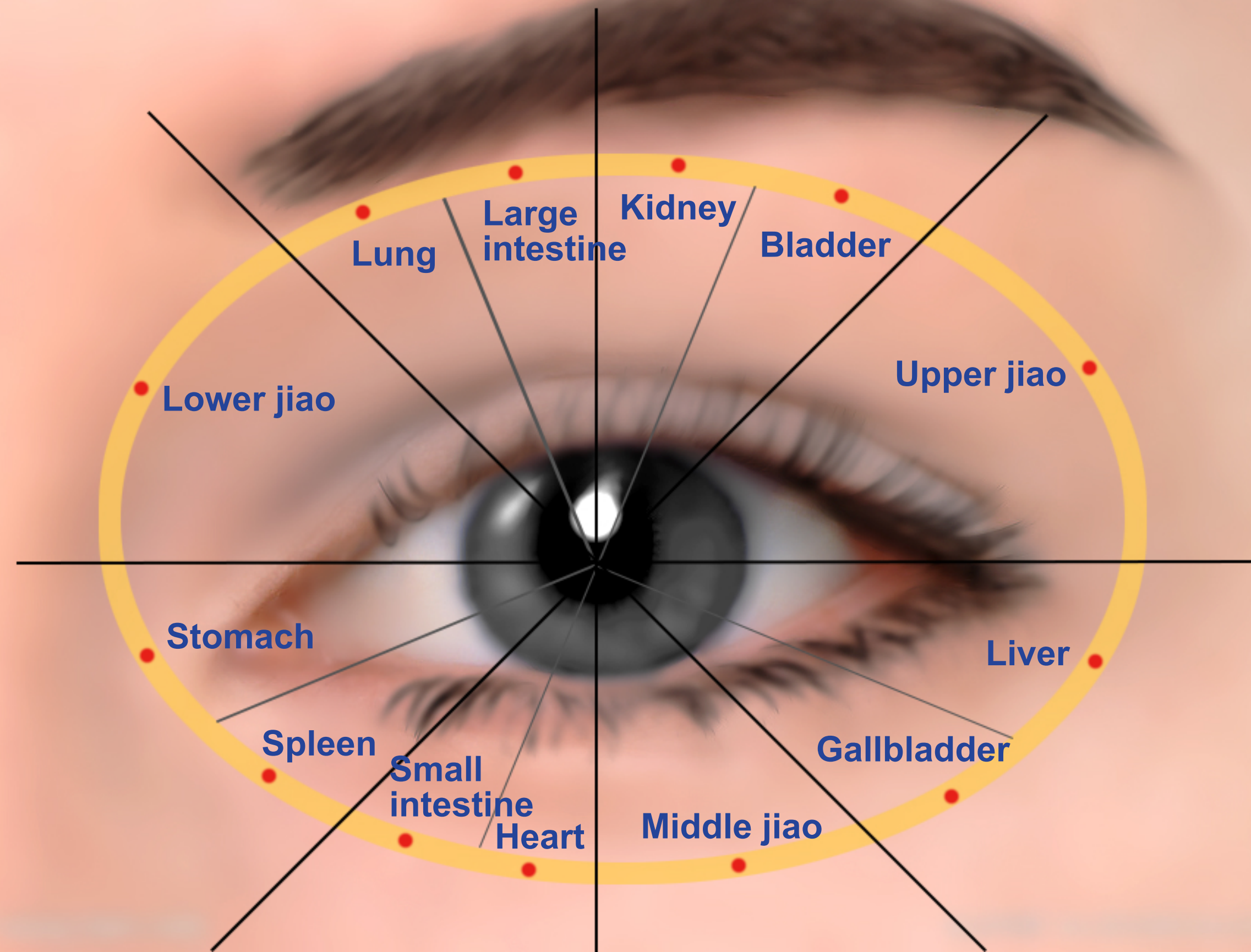

Supplement: Supplementary file 1 — Additional file 1. [file 12906_2021_3272_MOESM1_ESM.pdf]

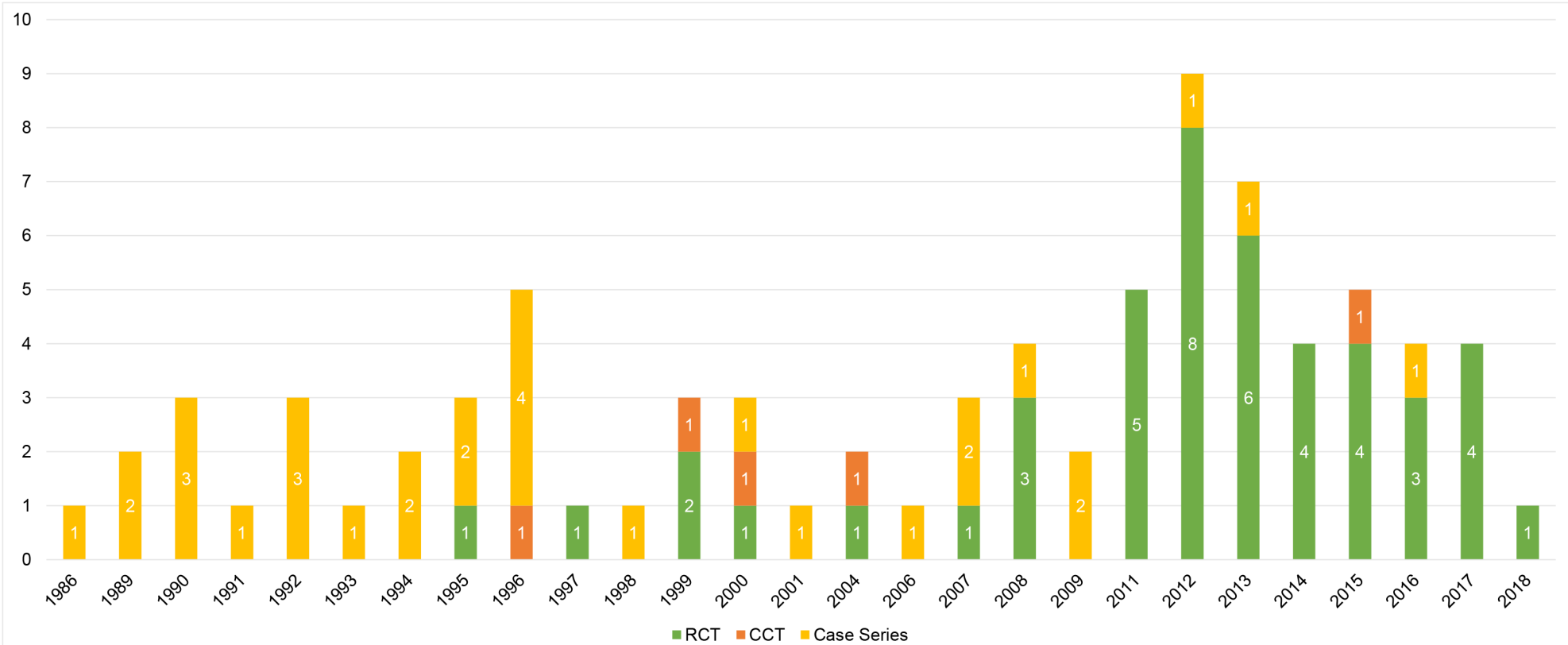

Supplement: Supplementary file 4 — Additional file 4. [file 12906_2021_3272_MOESM4_ESM.pdf]
