# Supplementary material for: Eye acupuncture for pain conditions: a scoping review of clinical studies
Source: BMC Complement Med Ther. 2021 Mar 23;21:101. doi: 10.1186/s12906-021-03272-8 (PMC7989101; doi:10.1186/s12906-021-03272-8)
Supplement: Supplementary file 2 — Additional file 2. [file 12906_2021_3272_MOESM2_ESM.pdf]

Additional file for "Eye Acupuncture for Pain Conditions: a Scoping Review of Clinical Studies"

Additional File 2. Search strategy and result for English database PubMed and Chinese database CNKI

PubMed search strategy and results:

Search	Query	Items found
#14	#13 AND ("1970"[Date - Publication] : "2018"[Date - Publication]	491
#13	#10 AND #11 AND #12	495
#12	#8 OR #9	800168
#11	#5 OR #6 OR #7	172962
#10	#1 OR #2 OR #3 OR #4	1020924
#9	"analgesia"[MeSH Terms] OR "analgesia"[All Fields]	80648
#8	"pain"[MeSH Terms] OR "pain"[All Fields]	768569
#7	"needles"[MeSH Terms] OR "needles"[All Fields] OR "needle"[All Fields] OR needling[All Fields]	147264
#6	"electroacupuncture"[MeSH Terms] OR "electroacupuncture"[All Fields]	4889
#5	"acupuncture"[MeSH Terms] OR "acupuncture"[All Fields] OR "acupuncture therapy"[MeSH Terms] OR ("acupuncture"[All Fields] AND "therapy"[All Fields]) OR "acupuncture therapy"[All Fields]	29632
#4	"eye sockets"[All Fields] OR"eye socket"[All Fields]	237
#3	"orbit"[MeSH Terms] OR "orbit"[All Fields] OR "orbits"[All Fields] OR orbital[All Fields] OR periorbital[All Fields] OR circumorbital[All Fields]	89010
#2	Optic[All Fields] OR optical[All Fields] OR ophthalmic[All Fields]	926591
#1	"eye"[MeSH Terms] OR "eye"[All Fields] OR "eyes"[All Fields]	642798

China Network Knowledge Infrastructure (CNKI) search strategy and results:

ID	Search	Hits
#1	[Subject]眼针	1075
#2	[Full text]痛	2805154
#3	#1 AND #2	377
#4	#3 AND 发表时间 between (1970-01-01,)	377
