# Supplementary material for: Eye acupuncture for pain conditions: a scoping review of clinical studies
Source: BMC Complement Med Ther. 2021 Mar 23;21:101. doi: 10.1186/s12906-021-03272-8 (PMC7989101; doi:10.1186/s12906-021-03272-8)
Supplement: Supplementary file 3 — Additional file 3. [file 12906_2021_3272_MOESM3_ESM.pdf]

Additional file for "Eye Acupuncture for Pain Conditions: a Scoping Review of Clinical Studies"

Additional file 3. Information of 30 case reports

We included 30 case reports[1-30] published between 1977 and 2016, conducted in China and Singapore (1, 3.3%), published in Chinese and English (1, 3.3%). Reports observed 58 patients aged 21 to 81, including 25 females (43.1%). A total of 54 conditions lasted from one day to ten years covered 29 categories (see Table), with acute lumbar sprain (7, 13.0%) and headache (5, 9.3%) being the most frequently ones. Only seven studies reported both length and diameter of the needles used and four of them chose φ0.30 mm × 13 mm. Eighteen studies applied eye acupuncture alone while the reminder used a combination of eye acupuncture with other interventions. All studies reported positive results which showed a 100% curative effect overall, with eight of them reported follow-ups from one day to one year. Immediate analgesic effect was detected in 46 (85.2%) conditions, and 18 conditions (33.3%) were treated with adjunctive exercise. Only one study used an instrument, the Visual Analogue Scale. No studies reported outcome of adverse event.

Table. Conditions, immediate analgesic effect and adjunctive exercise application (N=54)

Conditions	N, %	Immediate analgesic effect (n1, %)	adjunctive exercise (n2, %)
**Head, face, and mouth**	**12, 22.2%**	**8, 14.8%**	**-**
headache	5, 9.3%	4, 7.4%	-
trigeminal neuralgia	4, 7.4%	3, 5.6%	-
papillitis	1, 1.9%	-	-
facial pain	1, 1.9%	1, 1.9%	-
occipital neuralgia	1, 1.9%	-	-
**Upper shoulder and upper limbs**	**7, 13.0%**	**7, 13.0%**	**3, 5.6%**

periarthritis of shoulder	2, 3.7%	2, 3.7%	1, 1.9%
shoulder pain	2, 3.7%	1, 1.9%	-
scapular strain	1, 1.9%	1, 1.9%	1, 1.9%
chronic arm sprain	1, 1.9%	1, 1.9%	1, 1.9%
phantom limb pain	1, 1.9%	1, 1.9%	-
**Abdominal region**	**9, 16.7%**	**9, 16.7%**	**1, 1.9%**
biliary ascariasis	3, 5.6%	3, 5.6%	1, 1.9%
stomachache	2, 3.7%	2, 3.7%	-
acute cholecystitis	1, 1.9%	1, 1.9%	-
renal or ureteral colic	1, 1.9%	1, 1.9%	-
acute gastritis	1, 1.9%	1, 1.9%	-
acute gastroenteritis	1, 1.9%	1, 1.9%	-
**Lower back, lumbar spine, sacrum, and coccyx**	**13, 24.1%**	**13, 24.1%**	**8, 14.8%**
Acute lumbar sprain	7, 13.0%	7, 13.0%	7, 13.0%
lumbar disc herniation	3, 5.6%	2, 3.7%	-
chronic lumbar sprain	2, 3.7%	2, 3.7%	1, 1.9%
low back pain	1, 1.9%	1, 1.9%	-
**Lower limbs**	**7, 13.0%**	**6, 11.1%**	**5, 9.3%**
leg pain	2, 3.7%	2, 3.7%	1, 1.9%
ankle sprain	2, 3.7%	2, 3.7%	2, 3.7%
sciatica	1, 1.9%	1, 1.9%	1, 1.9%
knee joint degeneration	1, 1.9%	1, 1.9%	1, 1.9%
knee osteoarthritis	1, 1.9%	-	-
**Pelvic region**	**3, 5.6%**	**3, 5.6%**	**-**
dysmenorrhea	2, 3.7%	2, 3.7%	-
hypogastralgia	1, 1.9%	1, 1.9%	-
**Anal, perineal, and genital region**	**1, 1.9%**	**1, 1.9%**	**-**
acute orchitis	1, 1.9%	1, 1.9%	1, 1.9%
**Others**	**2, 3.7%**	**1, 1.9%**	**1, 1.9%**
acute gouty arthritis	2, 3.7%	1, 1.9%	1, 1.9%

References

1. Ma FQ, Yang ZB, Wang YD, Chen JL, Wang H: **Treatment of knee arthritis with eye acupuncture and body acupuncture**. *World Journal of Acupuncture-Moxibustion* 2016(04):72-74.
2. Dong LQ: **Summary of clinical experience of professor Zhang Mingbo in the treatment of disc-derived sciatica**. *Master.* Liaoning University of Traditional Chinese Medicine; 2015.
3. Chen YX: **Zhang Mingbo's clinical experience in the treatment of primary trigeminal neuralgia**. *Master.* Liaoning University of Traditional Chinese Medicine; 2015.
4. Liu ZD, Liang W: **The experience of treating lumbar disc herniation with lumbar compression fracture and scoliosis with eye acupuncture**. *International Journal of*

Traditional Chinese Medicine 2014(4):359-360.

5. Chen B: **Professor Wang pengqin's experience in the treatment of occipital neuralgia**. *Master.* Liaoning University of Traditional Chinese Medicine; 2014.
6. Xu HD: **Clinical application of eye acupuncture therapy in Singapore**. *Liaoning Journal of Traditional Chinese Medicine* 2013(11):2360-2362.
7. Xie WM, Hai Y: **Eye acupuncture for acute lumbar sprain by Professor Tian Weizhu**. *Journal of Practical Traditional Chinese Internal Medicine* 2013(14):22-23.
8. Wang F, Hai Y: **Eye acupuncture combined with three - hole acupuncture in the treatment of primary trigeminal neuralgia**. *Chinese Acupuncture & Moxibustion* 2013(1):99-101.
9. Gao ZG, Zhang MB: **Professor Zhang Ming-bo's experience of eye acupuncture combined with body acupuncture in the treatment of acute lumbar sprain**. *Journal of Liaoning University of Traditional Chinese Medicine* 2013(04):207-208.
10. Hai Y: **Professor Tian Weizhu's experience in treating diseases with eye acupuncture**. *World Chinese Medicine* 2012(02):114-115.
11. Xu F: **One case of acute gouty arthritis treated with eye acupuncture and intradermal needling**. In: *The 12th academic seminar of guangdong acupuncture society and the national training course on non-drug therapy for stroke and spinal correlating diseases: 2011; Zhongshan, Guangdong, China*; 2011: 2.
12. Wu ZL: **Acupuncture tfor knee joint pain: one case report**. In: *The 12th academic seminar of guangdong acupuncture society and the national training course on non-drug therapy for stroke and spinal correlating diseases: 2011; Zhongshan, Guangdong, China*; 2011: 1.
13. Jin L, Wang YG: **Phantom limb pain: a case report**. *Chinese Acupuncture & Moxibustion* 2011(11):1026.
14. He Y, Fu WB: **Professor Fu Wenbin's clinical experience of using eye acupuncture combined with opposing needling and long way acupuncture treating theacute gouty arthritis.** *Journal of Guangzhou University of Traditional Chinese Medicine* 2011(05):541-543.
15. Ding CY: **Professor Wang Pengqin's experience in the treatment of primary trigeminal neuralgia**. *Master.* Liaoning University of Traditional Chinese Medicine; 2011.
16. Chen FX: **The clinical experience of eye acupuncture therapy**. In: *Nongcun yiyao bao (han).* 2006: 002.
17. Tai XF: **The application of eye acupuncture in pain**. *Practical new medicine* 2001(10).
18. Liu XM, Ma CF: **Clinical application of eye acupuncture**. *Journal of Clinical Acupuncture and Moxibustion* 1996(04):28-29.
19. Fu WB: **Clinical application of eye acupuncture for acute lumbar sprain**. *Journal of Beijing University of Traditional Chinese Medicine* 1996(01):34.
20. Zhang YF: **Clinical application of eye acupuncture**. *Journal of External Therapy of Traditional Chinese Medicine* 1994, **3**(2):42-42.
21. Xie JF: **The application of eye acupuncture in pain**. *Jiangxi Journal of Traditional Chinese Medicine* 1994(5):44.
22. Zhou JN: **Clinical application of eye acupuncture**. *Journal of Clinical Acupuncture*

and Moxibustion 1993(06):26-27.

23. Meng B, Gao LZ: **Eye acupuncture for headache and dizziness**. *Journal of Sichuan of Traditional Chinese Medicine* 1993(05):52.
24. Yang SL: **Case records on eye acupuncture treatment**. *Journal of Sichuan of Traditional Chinese Medicine* 1991(12):50.
25. Wang SJ: **Two case records on eye acupuncture treatment**. *Liaoning Journal of Traditional Chinese Medicine* 1991(03):39-40.
26. Peng JS: **Selection of case records on eye acupuncture treatment**. *Liaoning Journal of Traditional Chinese Medicine* 1990(01):30-31.
27. Li HA: **Four case records on eye acupuncture treatment**. *Chinese Journal of Practical Medicine* 1989(05):43-44.
28. Li YX, Cao LH, Chen YF: **Treatment experience of eye acupuncture therapy**. *Chinese Archives of Traditional Chinese Medicine* 1988(03):38.
29. Han YB: **Two case records on eye acupuncture treatment**. *Modern Traditional Chinese Medicine* 1988(04):38-39.
30. Peng JS: **Selection of case records on eye acupuncture treatment**. *Liaoning Journal of Traditional Chinese Medicine* 1977(02):46-49.
