# Supplementary material for: Eye acupuncture for pain conditions: a scoping review of clinical studies
Source: BMC Complement Med Ther. 2021 Mar 23;21:101. doi: 10.1186/s12906-021-03272-8 (PMC7989101; doi:10.1186/s12906-021-03272-8)
Supplement: Supplementary file 5 — Additional file 5. [file 12906_2021_3272_MOESM5_ESM.pdf]

Additional file 5. Diseases/symptoms and acupoints (N = 110)

Diseases/symptoms	n, %	Top three used acupoints (m, %)
**Head, face, and mouth**	**19, 17.3%**	**Upper jiao (18, 17.8%), liver (17, 16.8%), kidney (12, 11.9%)**
headache	17, 15.5%	Upper jiao (16, 16.7%), liver (16, 16.7%), kidney (11, 11.5%)
trigeminal neuralgia	2, 1.8%	Upper jiao (2, 40.0%), liver (1, 20.0%), heart (1, 20.0%), kidney (1, 20.0%)
**Cervical region**	**5, 4.5%**	**Upper jiao (5, 29.4%), large intestine (4, 23.5%), small intestine (4, 23.5%)**
neck stiffness	3, 2.7%	Upper jiao (3, 33.3%), large intestine (2, 22.2%), small intestine (2, 22.2%), lung (2, 22.2%)
cervical spondylosis	2, 1.8%	Upper jiao (2, 25.0%), large intestine (2, 25.0%), small intestine (2, 25.0%)
**Upper shoulder and upper limbs**	**12, 10.9%**	**Upper jiao (12, 25.5%), lower jiao (5, 10.6%), liver (5, 10.6%), kidney (5, 10.6%)**
periarthritis of shoulder	5, 4.5%	Upper jiao (5, 50.0%), liver (3, 30.0%), gallbladder (2, 20.0%)
shoulder-hand syndrome after stroke	5, 4.5%	Upper jiao (5, 25.0%), lower jiao (3, 15.0%), heart (3, 15.0%), kidney (3, 15.0%)
shoulder pain	1, 0.9%	Upper jiao (1, 7.7%), middle jiao (1, 7.7%), lower jiao (1, 7.7%), large intestine (1, 7.7%), small intestine (1, 7.7%), heart (1, 7.7%), liver (1, 7.7%), lung (1, 7.7%), kidney (1, 7.7%), stomach (1, 7.7%), gallbladder (1, 7.7%), bladder (1, 7.7%), spleen (1, 7.7%)
lateral humeral epicondylitis	1, 0.9%	Upper jiao (1, 25.0%), lower jiao (1, 25.0%), liver (1, 25.0%), kidney (1, 25.0%)

Additional file for "Eye Acupuncture for Pain Conditions: a Scoping Review of Clinical Studies"

**Thoracic region**	**2, 1.8%**	**Liver (2, 20.0%), gallbladder (2, 20.0%), stomach (1, 10.0%), upper jiao (1, 10.0%), lower jiao (1, 10.0%), heart (1, 10.0%), spleen (1, 10.0%), kidney (1, 10.0%)**
heart plant nerve function disorder	1, 0.9%	Upper jiao (1, 16.7%), lower jiao (1, 16.7%), heart (1, 16.7%), liver (1, 16.7%), kidney (1, 16.7%), gallbladder (1, 16.7%)
chronic mammary gland hyperplasia	1, 0.9%	Liver (1, 25.0%), stomach (1, 25.0%), gallbladder (1, 25.0%), spleen (1, 25.0%)
**Abdominal region**	**21, 19.1%**	**Middle jiao (14, 14.4%), liver (14, 14.4%), gallbladder (13, 13.4%)**
irritable bowel syndrome	4, 3.6%	Large intestine (4, 22.2%), spleen (4, 22.2%), lower jiao (3, 16.7%), liver (3, 16.7%)
biliary colic	4, 3.6%	Gallbladder (4, 40.0%), middle jiao (3, 30.0%), liver (2, 20.0%)
renal or ureteral colic	3, 2.7%	Lower jiao (3, 27.3%), middle jiao (1, 9.1%), large intestine (1, 9.1%), small intestine (1, 9.1%), liver (1, 9.1%), kidney (1, 9.1%), stomach (1, 9.1%), gallbladder (1, 9.1%), bladder (1, 9.1%)
acute cholecystitis	3, 2.7%	Gallbladder (3, 20.0%), middle jiao (2, 20.0%), liver (2, 20.0%)
biliary ascariasis	2, 1.8%	Gallbladder (2, 22.2%), middle jiao (2, 22.2%), lower jiao (1, 11.1%), large intestine (1, 11.1%), small intestine (1, 11.1%), liver (1, 11.1%), stomach (1, 11.1%)
achalasia	1, 0.9%	Middle jiao (1, 20.0%), liver (1, 20.0%), kidney (1, 20.0%), spleen (1, 20.0%), stomach (1, 20.0%)
acute gastroenteritis	1, 0.9%	Middle jiao (1, 14.3%), lower jiao (1, 14.3%), large intestine (1, 14.3%), small intestine (1,

		14.3%), liver (1, 14.3%), stomach (1, 14.3%), gallbladder (1, 14.3%)
stomachache	1, 0.9%	Middle jiao (1, 7.7%), upper jiao (1, 7.7%), lower jiao (1, 7.7%), large intestine (1, 7.7%), small intestine (1, 7.7%), heart, liver (1, 7.7%), lung (1, 7.7%), kidney (1, 7.7%), stomach (1, 7.7%), gallbladder (1, 7.7%), bladder (1, 7.7%), spleen (1, 7.7%)
acute pancreatitis	1, 0.9%	Lower jiao (1, 14.3%), middle jiao (1, 14.3%), large intestine (1, 14.3%), small intestine (1, 14.3%), liver (1, 14.3%), stomach (1, 14.3%), gallbladder (1, 14.3%)
duodenal ulcer	1, 0.9%	Lower jiao (1, 14.3%), middle jiao (1, 14.3%), large intestine (1, 14.3%), small intestine (1, 14.3%), liver (1, 14.3%), stomach (1, 14.3%), gallbladder (1, 14.3%)
**Lower back, lumbar spine, sacrum, and coccyx**	**28, 25.5%**	**Lower jiao (26, 30.2%), kidney (16, 18.6%), liver (9, 10.5%), bladder (9, 10.5%)**
acute lumbar sprain	7, 6.4%	Lower jiao (6, 46.2%), kidney (3, 23.1%), upper jiao (1, 7.7%), liver (1, 7.7%), gallbladder (1, 7.7%), bladder (1, 7.7%)
lumbar disc herniation	7, 6.4%	Lower jiao (6, 35.3%), kidney (4, 23.5%), bladder (4, 23.5%)
low back pain	4, 3.6%	Lower jiao (4, 14.8%), liver (4, 14.8%), kidney (4, 14.8%)
lumbar spinal stenosis	2, 1.8%	Lower jiao (2, 40.0%), middle jiao (1, 20.0%), liver (1, 20.0%), kidney (1, 20.0%)
lumbar muscle degeneration	2, 1.8%	Lower jiao (2, 33.3%), upper jiao (1, 16.7%), liver (1, 16.7%), kidney (1, 16.7%), gallbladder (1, 16.7%)

chronic lumbar sprain	1, 0.9%	Lower jiao (1, 20.0%), kidney (1, 20.0%), upper jiao (1, 20.0%), liver (1, 20.0%), gallbladder (1, 20.0%)
third lumbar transverse process syndrome	1, 0.9%	Lower jiao (1, 100%)
lumbar osteoarthritis	1, 0.9%	Lower jiao (1, 50.0%), middle jiao (1, 50.0%)
traumatic low back pain	1, 0.9%	Lower jiao (1, 25.0%), middle jiao (1, 25.0%), bladder (1, 25.0%), kidney (1, 25.0%)
lumbar hyperosteogeny	1, 0.9%	Lower jiao (1, 20.0%), upper jiao (1, 20.0%), liver (1, 20.0%), kidney (1, 20.0%), gallbladder (1, 20.0%)
supraspinal ligament injury	1, 0.9%	Lower jiao (1, 100%)
**Lower limbs**	**8, 7.3%**	**Lower jiao (8, 21.1%), kidney (6, 15.8%), gallbladder (5, 13.2%), bladder (5, 13.2%)**
sciatica	5, 4.5%	Lower jiao (5, 31.3%), gallbladder (4, 25.0%), bladder (4, 25.0%) kidney (3, 18.8%)
leg pain	1, 0.9%	Middle jiao (1, 7.7%), upper jiao (1, 7.7%), lower jiao (1, 7.7%), large intestine (1, 7.7%), small intestine (1, 7.7%), heart, liver (1, 7.7%), lung (1, 7.7%), kidney (1, 7.7%), stomach (1, 7.7%), gallbladder (1, 7.7%), bladder (1, 7.7%), spleen (1, 7.7%)
ankle sprain	1, 0.9%	Lower jiao (1, 25.0%), upper jiao (1, 25.0%), liver (1, 25.0%), kidney (1, 25.0%)
thromboangiitis obliterans	1, 0.9%	Lower jiao (1, 20.0%), liver (1, 20.0%), lung (1, 20.0%), kidney (1, 20.0%), spleen (1, 20.0%)
**Pelvic region**	**5, 4.5%**	**Lower jiao (5, 21.7%), kidney (5, 21.7%), liver (5, 21.7%)**

dysmenorrhea	5, 4.5%	Lower jiao (5, 21.7%), kidney (5, 21.7%), liver (5, 21.7%)
**Anal, perineal, and genital region**	**1, 0.9%**	**Lower jiao (1, 100%)**
post-hemorrhoidectomy pain	1, 0.9%	Lower jiao (1, 100%)
**Others**	**9, 8.2%**	**Lower jiao (8, 33.3%), upper jiao (7, 16.7%), liver (7, 16.7%)**
acute gouty arthritis	2, 1.8%	Lower jiao (2, 40.0%), upper jiao (2, 40.0%), heart (1, 20.0%)
herpes zoster	2, 1.8%	Upper jiao (2, 11.8%), middle jiao (2, 11.8%), lower jiao (2, 11.8%), liver (2, 11.8%), lung (2, 11.8%), gallbladder (2, 11.8%), spleen (2, 11.8%)
neck-shoulder-back pain	1, 0.9%	Upper jiao (1, 25.0%), lower jiao (1, 25.0%), liver (1, 25.0%), kidney (1, 25.0%)
limbs pain	1, 0.9%	Upper jiao (1, 25.0%), lower jiao (1, 25.0%), liver (1, 25.0%), kidney (1, 25.0%)
military training injury	1, 0.9%	Upper jiao (1, 25.0%), lower jiao (1, 25.0%), liver (1, 25.0%), kidney (1, 25.0%)
thalamic pain syndrome	1, 0.9%	Liver (1, 33.3%), gallbladder (1, 33.3%), spleen (1, 33.3%)
sicca syndrome related arthralgia	1, 0.9%	Lower jiao (1, 20.0%), middle jiao (1, 20.0%), liver (1, 20.0%), kidney (1, 20.0%), spleen (1, 20.0%)

This table shows the top three reported frequency and percentage of acupoints by each disease/symptom.

m=frequency the acupoints used; %=m/total frequency of all the acupoints used in that condition

Others: diseases/symptoms contain more than one region
