# Supplementary material for: Eye acupuncture for pain conditions: a scoping review of clinical studies
Source: BMC Complement Med Ther. 2021 Mar 23;21:101. doi: 10.1186/s12906-021-03272-8 (PMC7989101; doi:10.1186/s12906-021-03272-8)
Supplement: Supplementary file 6 — Additional file 6. [file 12906_2021_3272_MOESM6_ESM.pdf]

Additional file 6. Details of interventions in each comparison

Intervention group	N	Control group	n
**Eye acupuncture**	**22**		
		Non-pharmaceutical TCM[1]	10
		Conventional medicine[2]	9
		Pharmaceutical TCM[3]	3
**Eye acupuncture + Non-pharmaceutical TCM**	**12**		
		Non-pharmaceutical TCM	7
		Conventional medicine	3
		Pharmaceutical TCM + Conventional medicine	1
		Pharmaceutical TCM + Non-pharmaceutical TCM	1
**Eye acupuncture + Pharmaceutical TCM**	**5**		
		Pharmaceutical TCM	3
		Conventional medicine	2
**Eye acupuncture + Conventional treatment**	**3**		
		Conventional medicine + Conventional treatment[4]	3
**Eye acupuncture + Non-pharmaceutical TCM + Non-pharmaceutical conventional therapy + Conventional treatment**	**3**		
		Non-pharmaceutical conventional therapy[5] + Conventional treatment	2
		Non-pharmaceutical TCM + Non-pharmaceutical conventional therapy + Conventional treatment	1
**Eye acupuncture + Non-pharmaceutical TCM + Non-pharmaceutical conventional therapy**	**2**		

		Conventional medicine + Non-pharmaceutical conventional therapy	1
		Non-pharmaceutical conventional therapy	1
**Eye acupuncture + Non-pharmaceutical conventional therapy**	**2**		
		Non-pharmaceutical TCM + Non-pharmaceutical conventional therapy	1
		Non-pharmaceutical conventional therapy	1
**Eye acupuncture + Non-pharmaceutical conventional therapy + Conventional treatment**	**2**		
		Non-pharmaceutical conventional therapy + Conventional treatment	2
**Eye acupuncture + Non-pharmaceutical TCM + Conventional treatment**	**1**		
		Conventional medicine + Conventional treatment	1
**Eye acupuncture + Non-pharmaceutical TCM + Pharmaceutical TCM + Conventional treatment**	**1**		
		Non-pharmaceutical TCM + Pharmaceutical TCM + Conventional treatment	1
**Eye acupuncture + Pharmaceutical TCM + Conventional medicine + Non-pharmaceutical TCM + Conventional treatment**	**1**		
		Pharmaceutical TCM + Conventional medicine + Non-pharmaceutical TCM + Conventional treatment	1
**Eye acupuncture + Non-pharmaceutical TCM + Pharmaceutical TCM**	**1**		
		Non-pharmaceutical TCM + Pharmaceutical TCM	1

Note: interventions reported in each category

1 Non-pharmaceutical TCM: body acupuncture, acupuncture at forearm reaction point, Fu acupuncture, scalp acupuncture, locality surrounding acupuncture at skin lesion area, encircling needling, Canggui Tanxue needling, locality ironing therapy, Tuina, thumb pushing manipulation, blood-letting, cupping and Leihuo moxibustion

2 Conventional medicine (analgesic drugs in generic name): Ibuprofen, Flunarizine Hydrochloride, Atropine, Morphine, Bucinnazine Hydrochloride, Pinaverium Bromide and Indomethacin

3 Pharmaceutical TCM: compound Chinese medicine decoration, Chinese patent medicine and external application with Chinese medicine

4 Conventional treatment: basic treatment or symptomatic treatment such as antihypertensive treatment, anti-inflammatory, analgesic treatment, neurotrophic drug treatment, etc., varied in different diseases.

5 Non-pharmaceutical conventional therapy: polarized light therapy, rehabilitation therapy, lumbar traction, semiconductor laser irradiation and physiotherapy.
